# Supplementary figures and images for: An Allelopathic Role for Garlic Root Exudates in the Regulation of Carbohydrate Metabolism in Cucumber in a Hydroponic Co-Culture System
Source: Plants (Basel). 2019 Dec 27;9(1):45. doi: 10.3390/plants9010045 (PMC7020217; doi:10.3390/plants9010045)

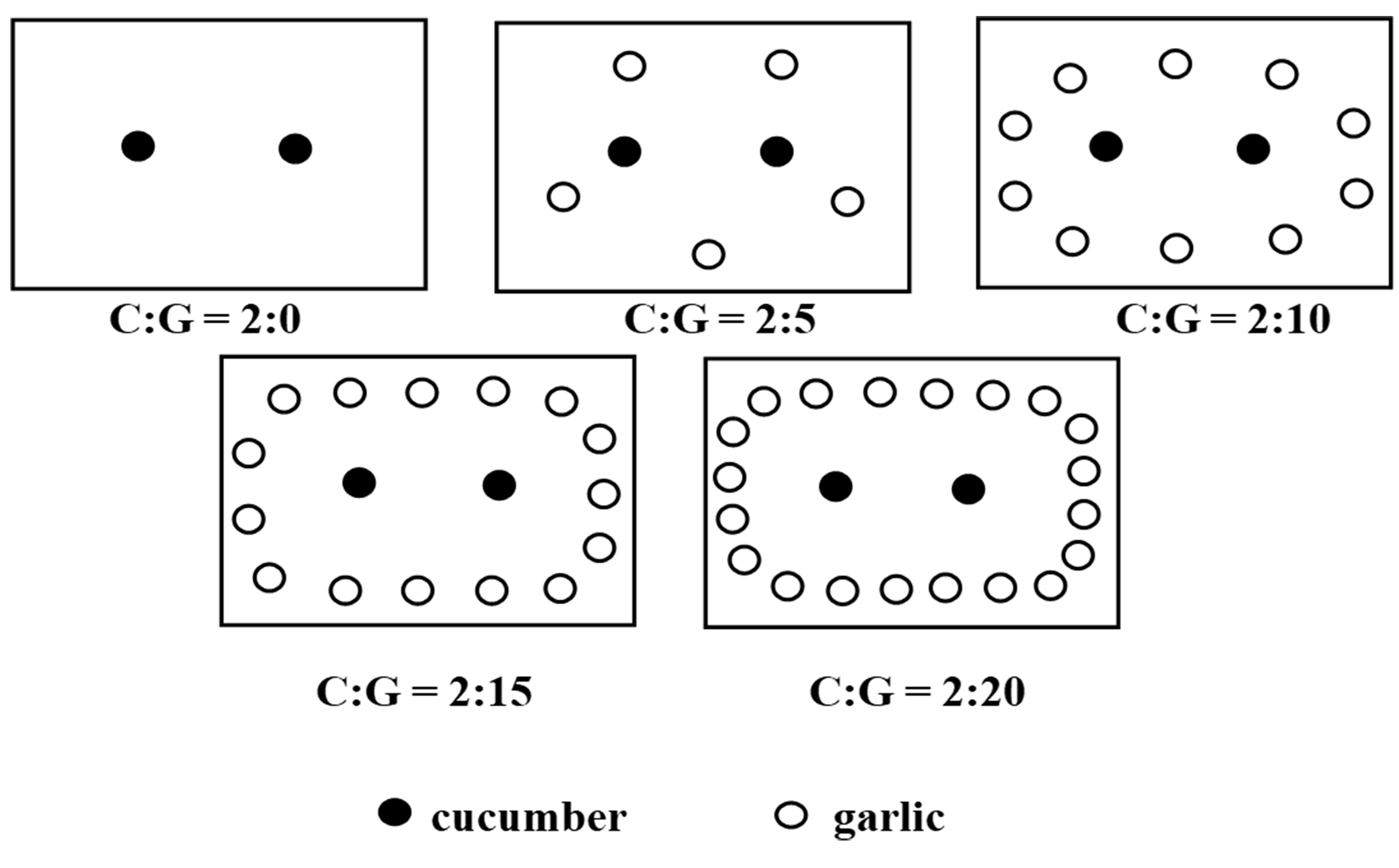

Supplement: Supplementary file 1 [file plants-09-00045-s001.tif]
